# Management of a Complex Crown‐Root Fracture in a Single Appointment Through Root Canal Therapy and Rebonding

**DOI:** 10.1002/ccr3.70332

**Published:** 2025-03-17

**Authors:** Mohammed H. AbdElaziz, Meshal Alharbi, Maher O. Shahada, Roqia Abdoh, Radhwan Saleh Algabri, Ahmed Yaseen Alqutaibi

**Affiliations:** ^1^ Substitutive Dental Science Department, College of Dentistry Taibah University Madinah Saudi Arabia; ^2^ Fixed Prosthodontics Department, Faculty of Dental Medicine Al‐Azhar University Cairo Egypt; ^3^ Resident in Endodontics Department, Specialized Dental Centre, Ministry of Health King Fahad Hospital Madinah Saudi Arabia; ^4^ Program Director of Post Graduated in Prosthodontics, Ministry of Health King Fahad Hospital, Specialized Dental Center Madinah Saudi Arabia; ^5^ Prosthodontic Department, Faculty of Dentistry Ibb University Ibb Yemen; ^6^ Department of Prosthodontics, Faculty of Dentistry National University Ibb Yemen; ^7^ Department of Prosthodontics, Faculty of Dentistry Ibb University Ibb Yemen

**Keywords:** biological width, crown reattachment, crown‐root fracture, fiber post

## Abstract

A 10‐year‐old male presented with a mobile and fractured maxillary right central incisor following dental trauma. Clinical and radiographic examinations revealed a chisel‐type crown‐root fracture with supragingival labial and subgingival palatal extensions. A direct crown reattachment was planned, emphasizing biological width preservation. After removing the coronal fragment, endodontic treatment was performed using a rotary file system and was obturated with a bioceramic sealer. A fiber post was selected and integrated into the fractured fragment, which was reattached using resin cement. Additionally, a gingivectomy was performed to enhance margin visibility and restore biological width. The adjacent left central incisor was treated for an uncomplicated crown fracture using follow‐up evaluations over 12 months revealed stable fragment reattachment, good periodontal health, and restored aesthetics and function. A custom mouthguard was provided to prevent future trauma. This case highlights the efficacy of a multidisciplinary approach combining endodontic, restorative, and periodontal techniques for managing crown‐root fractures, achieving favorable long‐term outcomes in pediatric patients.

**Summary**

- A multidisciplinary approach involving endodontic, restorative, and periodontal techniques can effectively manage crown‐root fractures in pediatric patients, ensuring preservation of biological width, stable fragment reattachment, periodontal health, and restored aesthetics and function.

## 1 Introduction

Facial injuries exhibit considerable diversity, with dentoalveolar trauma ranking among the most prevalent types [1]. Within this classification, crown fractures—whether or not they involve pulp exposure—are the most frequently observed, constituting between 26.2% and 44.1% of all dental injuries [2]. In contrast, crown‐root fractures, irrespective of pulp involvement, are comparatively rare, accounting for only 0.56%–1.1% of dental injuries [2]. A sudden impact to a tooth and its surrounding tissues can result in a variety of injuries, including complete crown‐root fractures, which compromise the enamel, dentin, cementum, and pulp, often extending subgingivally [3, 4]. Among dental trauma cases, maxillary incisors are the most commonly affected teeth, with crown fractures predominating [5].

The management of complicated crown‐root fractures presents significant challenges, particularly due to the difficulties associated with maintaining adequate isolation using a rubber dam. Such isolation is essential for creating a dry working environment and achieving a hermetic seal, both of which are critical for the success of the treatment [6]. Treatment modalities for anterior teeth with complicated crown‐root fractures vary based on factors such as the fracture's relation to the alveolar crest, the degree of pulpal involvement, the extent of root apex formation, and the patient's aesthetic requirements [7]. The primary objectives are to prevent tooth loss and to restore both function and aesthetics, with particular attention given to the gingival attachment apparatus to avoid soft tissue deformities. These deformities frequently arise when the biological width is compromised during the restoration of defects [5]. For mature teeth with complete root formation, pulp removal is typically indicated [8].

Treatment options for dental trauma encompass a spectrum of interventions, including root canal therapy, restoration, orthodontic extrusion, surgical extrusion, root submersion, intentional replantation, extraction, and autotransplantation [8]. Recent advancements in restorative materials and techniques have rendered the reattachment of fractured teeth a viable and effective treatment modality [7]. This approach presents several advantages, such as immediate restoration of aesthetic appearance, morphology, and function; achievement of lifelike translucency; preservation of occlusal contacts; reduction of chairside time; and positive psychological effects [8]. Moreover, reattachment is characterized by its minimally invasive nature, cost‐effectiveness, and rapid restoration of speech, function, and appearance [8]. The significance of conservative treatment is particularly pronounced in younger patients, as it aids in the preservation of alveolar bone height, thereby ensuring future options for surgical and prosthetic rehabilitation [3].

The introduction of tooth‐colored fiber posts in the 1990s marked a notable advancement in treatment methodologies, providing advantages such as enhanced aesthetics, robust bonding with tooth structure, and dentin‐like elasticity [7]. Nevertheless, the implementation of fiber posts necessitates dentin preparation to facilitate proper fit [7].

This article delineates a comprehensive treatment plan for crown‐root fractures that extend below the gumline, with an emphasis on simplicity, aesthetic considerations, and the preservation of tooth structure in the context of managing cases of dental trauma.

## 2 Case History/Examination

**FIGURE 1:**
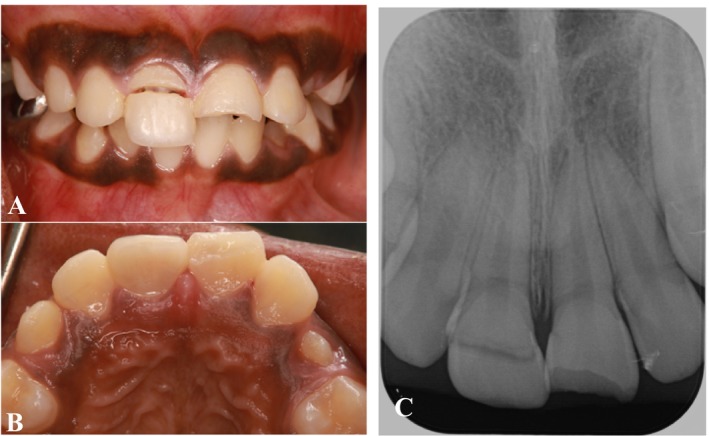
Preoperative clinical photos and radiograph. (A) Frontal view. (B) Maxillary occlusal view. (C) Preoperative periapical radiograph.

A 10‐year‐old male patient presented to the Dental College and Hospital at Taibah University Hospital, Dental Department, Madinah, Saudi Arabia, with complaints of mobility and fracture of teeth in the maxillary anterior region. The patient reported having sustained trauma 2 days prior to presentation. His medical history was unremarkable. Upon conducting extraoral and intraoral examinations, no significant trauma to the soft tissues was observed. Through clinical and radiographic evaluations (Figure 1), a diagnosis of a chisel‐type crown‐root fracture of the maxillary right central incisor was established.

**FIGURE 2:**
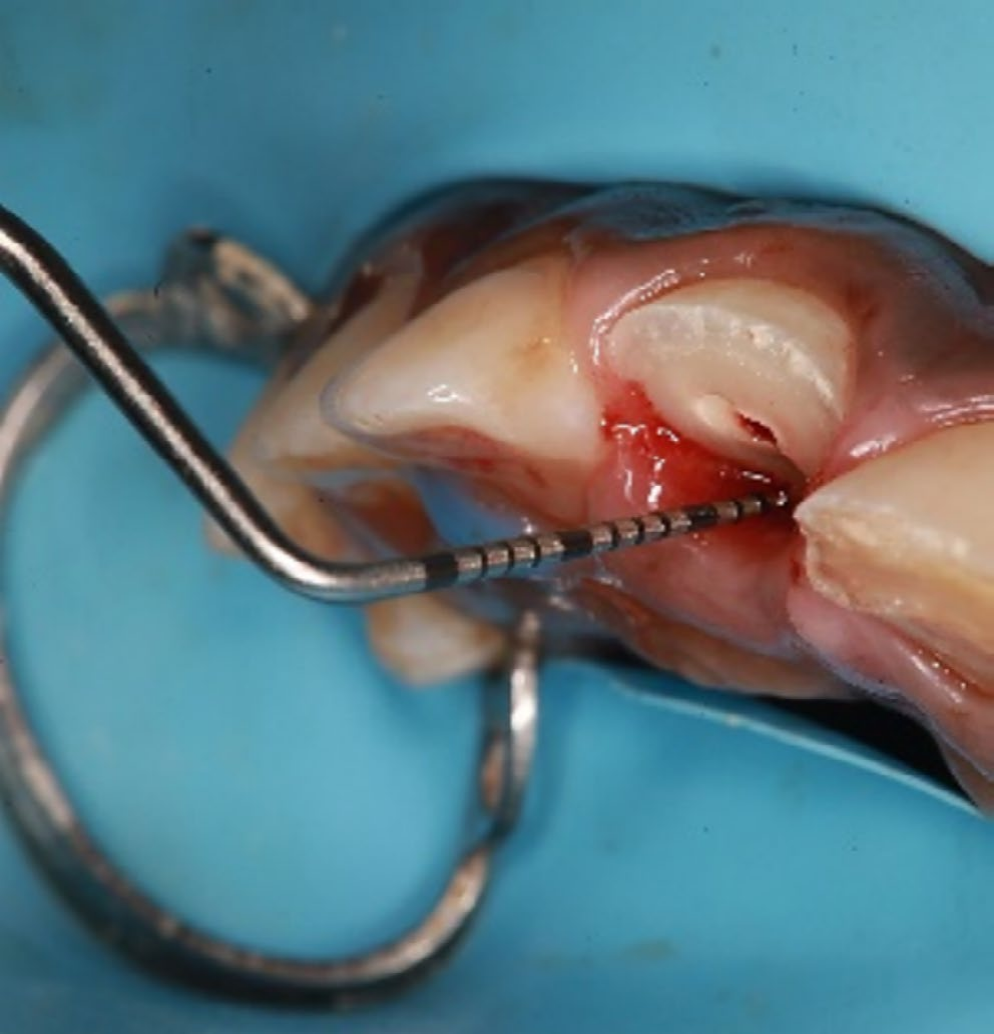
Measuring the biological width by using the periodontal UNC‐15 probe.

The fracture lines of the tooth were observed to be supragingival on the labial aspect and subgingival on the palatal aspect. To determine the biological width, we measured the probing depth and performed intrasulcular bone sounding following the administration of local anesthesia. The probing depth was measured at 3 mm on the palatal side (Figure 2). Notably, the palatal gingiva and interdental papilla exhibited signs of inflammation and edema. Additionally, there was no evident periapical pathosis (Figure 1).

## 3 Methods

**FIGURE 3:**
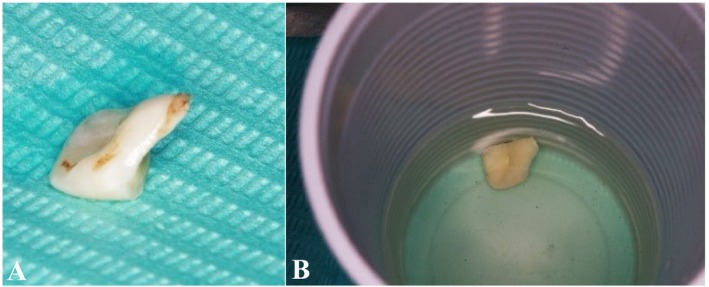
(A) Fractured coronal fragment. (B) Stored the fragment in a saline solution.

We devised a treatment plan for the direct reattachment of the crown fragment and engaged in a comprehensive discussion regarding this plan with the patient and the patient's parent, who subsequently provided informed consent. Following this, we meticulously excised the fractured coronal fragment while ensuring no inadvertent damage occurred (Figure 3). Subsequently, the pulp chamber within the coronal fracture fragment was thoroughly cleaned and preserved in saline to prevent discoloration and dehydration (Figure 3).

**FIGURE 4:**
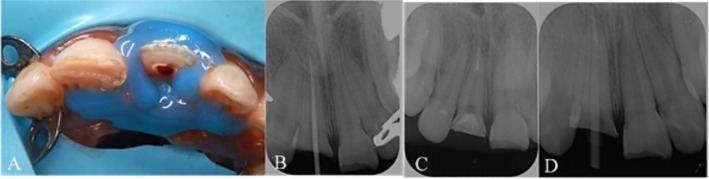
(A) Maxillary occlusal view. (B) Master cone fit. (C) Obturation with post space. (D) Post fit.

Challenges were encountered in the application of the rubber dam and clamp on the tooth, which necessitated the implementation of the split dam technique in conjunction with a liquid dam (DentAct Aqua Dam Gingival Barrier, DentAct, India) to achieve optimal isolation (Figure 4A).

Subsequently, the remaining pulp tissue within the root canal was extirpated using a barbed broach file (DentSply Pre‐Sterilized Endodontic Barbed Broaches, Dentsply Sirona USA). The working length was ascertained with the aid of an apex locator (J. Morita Root ZX II Apex Locator Canal Measurement, Japan) and subsequently confirmed through periapical radiography. The canal was then cleaned and shaped utilizing a rotary file (Dentsply Pro Taper Gold Endodontic Rotary File, USA) and irrigation by sodium hypochlorite 5.25% in conjunction with a crown‐down technique. Following this, the dryness was assessed by a paper point (Dentsply Pro Taper Conform Fit paper point, USA), and the master cone (Dentsply Pro Taper Conform Fit Gutta percha, USA) was selected and confirmed via an intraoral periapical radiograph (Figure 4B), and the canal was obturated employing the single cone technique (Dentsply Pro Taper Conform Fit Gutta percha, USA) in combination with a bioceramic sealer (Ceraseal Calcium Silicate Based Bio‐Ceramic Sealer, Korean).

System B (Woodpecker endo obturation system, China) was utilized to trim the gutta‐percha to the specified post space level, which was determined to be 5 mm apically. Thereafter, a post drill (3M ESPE RelyX Fiber Post Dental Drill size 2, 1.6 mm Red, USA) was employed to prepare the post space, and a radiograph was obtained to evaluate the adequacy of the space (Figure 4C). Finally, a fiber post (3M ESPE Dental RelyX Glass Fiber Post size 2, 1.6 mm Red, USA) was selected, and an additional radiograph was taken to ensure the proper fit of the fiber post (Figure 4D).

**FIGURE 5:**
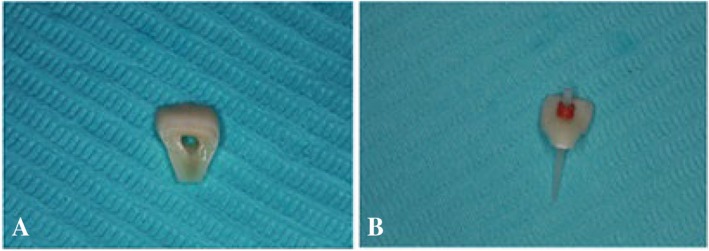
Fractured segment. (A) Opening the access through the fractured segment. (B) Insert the fiber post through the fractured segment.

**FIGURE 6:**
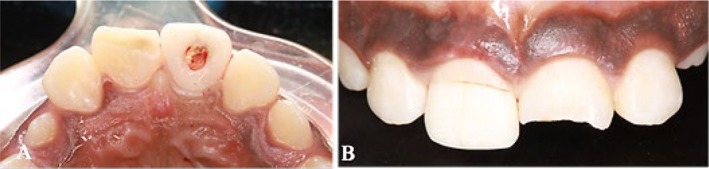
(A) Maxillary occlusal view. (B) Frontal view.

Subsequently, access was obtained through the fractured fragment (Figure 5A), and the fiber post was inserted through this access point (Figure 5B). Following this, a try‐in procedure was conducted to ensure the proper fit of the fractured fragment and post within the tooth (Figure 6).

**FIGURE 7:**
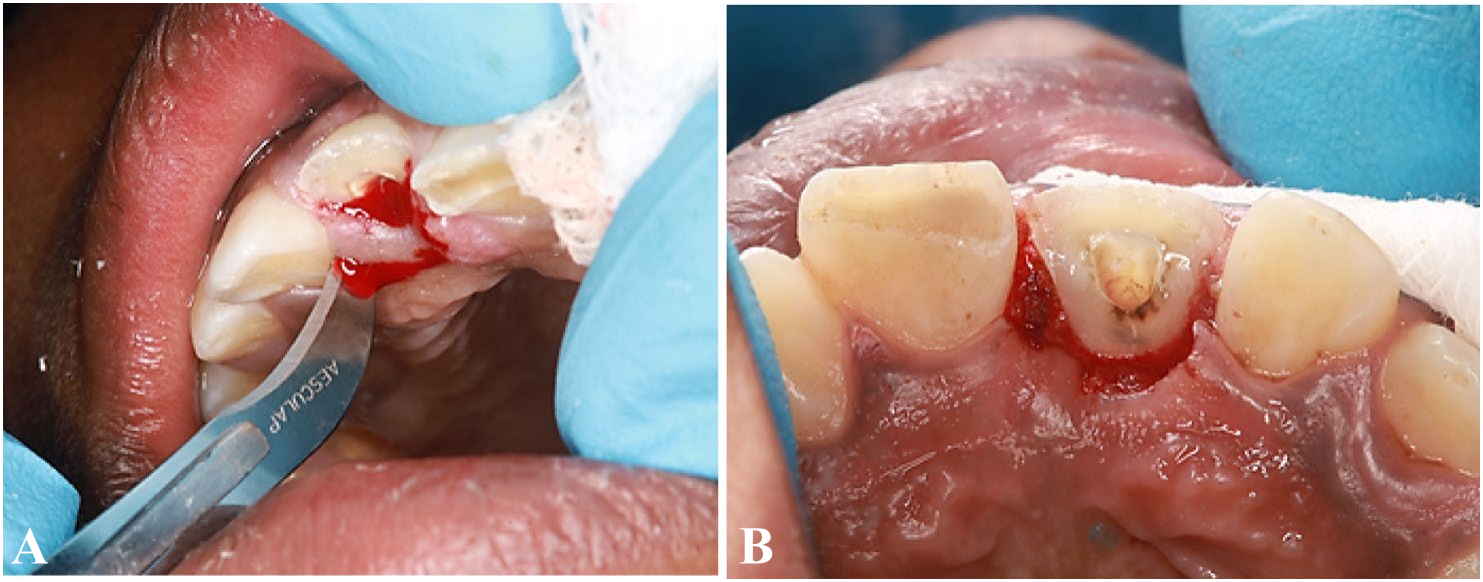
(A) Gingivectomy by using blade no. 12. (B) after removing 2 mm of soft tissue.

**FIGURE 8:**
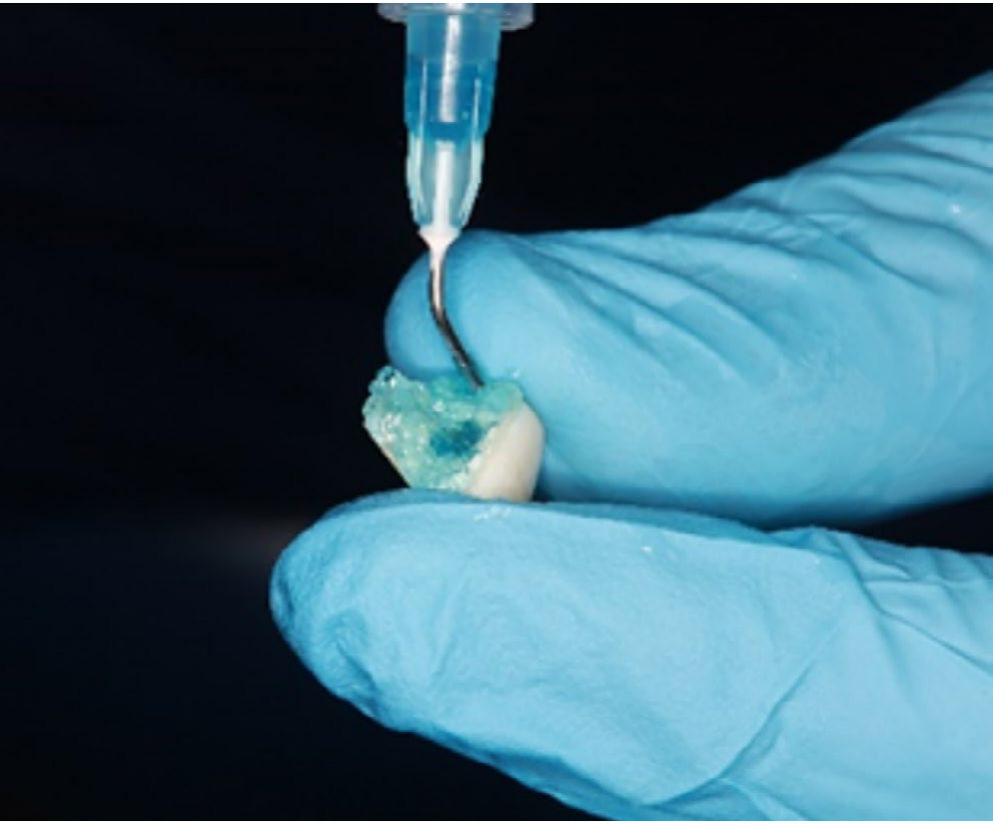
Etching the fractured fragment.

**FIGURE 9:**
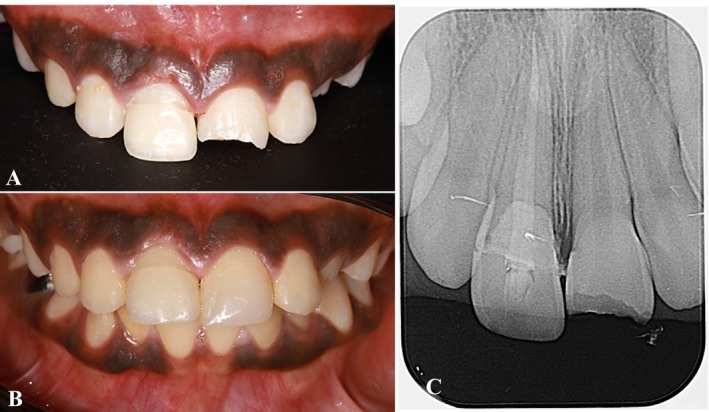
Post‐operative clinical photos and radiograph (A) Frontal view after cementing the fractured fragment. (B) Frontal view after restoring tooth #21 by resin‐bonded composite restoration. (C) Periapical radiograph after cementation.

A periodontist subsequently performed an external bevel gingivectomy, marking the depth of the sulcus with a pocket marker (Periodontal Pocket Markers Goldman Fox, India). A No. 12 surgical blade was utilized to excise 2 mm of the inflamed and edematous gingiva palatally, thereby exposing the margins while concurrently maintaining the biological width (Figure 7). Following this, the fractured area was etched using a 37% phosphoric acid solution (ANY‐ETCH 37% Phosphoric acid etching agent, South Korea), through a selective etching technique, with etching times of 30 s for enamel and 15 s for dentin. The surface was then rinsed with water using a cotton pellet (Figure 8). Hemostasis was achieved, after which the surface of the canal was etched with 37% phosphoric acid for an additional 15 s. The surface was again rinsed with water, and dried with air and a paper point, after which a bonding agent (3M ESPE Single Bond Universal Bonding Adhesive, USA) was applied using a microtip brush (Dental Micro Applicator Brush Bendable Superfine). This was followed by light curing for 5 s. Subsequently, resin cement (Calibra Ceram, Resin Cement, Dual Cure Automix, USA) was applied to the surface of the post and within the post preparation, and cured (light‐emitting diode curing light, China; Figure 9). Isolation was maintained throughout the procedure using cotton rolls and a saliva ejector.

The fragment was then reattached to the tooth, and excess cement along the margins was removed with a No. 12 blade and dental floss. The material was light cured using a light‐emitting diode curing light for 40 s from both buccal and palatal directions. The tooth surface was polished (Figure 9). Additionally, the maxillary left central incisor, which exhibited an uncomplicated crown fracture, was restored using a resin‐bonded composite restoration (Ivoclar Tetric N Ceram Light‐Curing, Radiopaque Nano‐Hybrid Composite. Liechtenstein; Figure 9).

**FIGURE 10:**
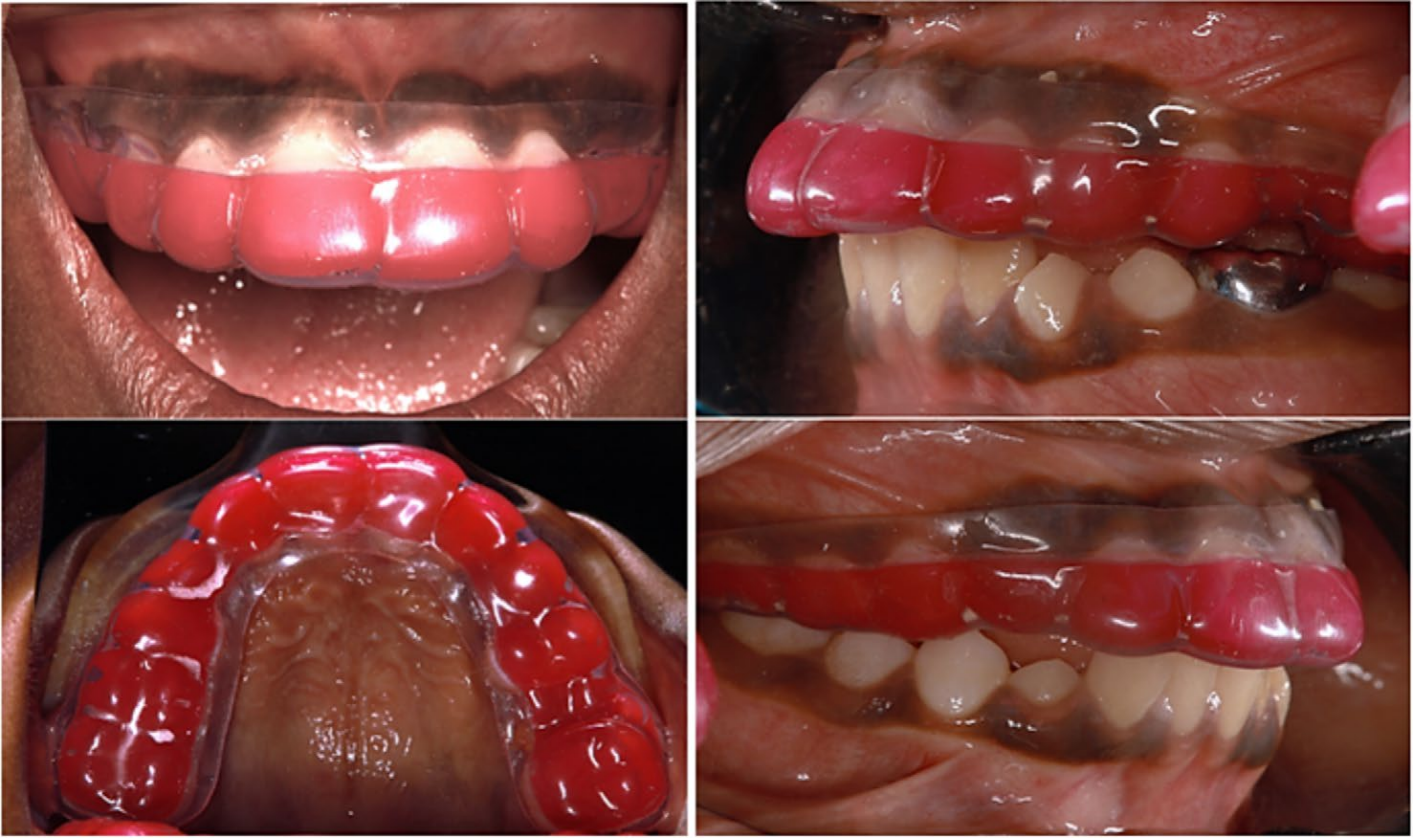
Clinical photos after placement of sporty mouth guard.

The patient presented with an increasing overjet of approximately 4 mm. Consequently, we advised the parent to consult an orthodontist for further evaluation. In the interim, we fabricated a sports mouthguard (Thermoplastic EVA polymer) to safeguard the patient's dentition, offering three color options—yellow, green, and red—of which the patient selected red (Figure 10). Postoperative instructions were given, which include:

- Clean after each use: rinse your mouthguard thoroughly with cool water immediately after use.
- Dry completely: this helps prevent bacteria and mold growth.
- Proper storage: avoid storing it in a sealed or moist environment.
- Avoid heat and sunlight: keep the mouth guard away from direct sunlight, hot surfaces, or inside a hot car, as excessive heat can warp the material.
- Inspect regularly: check your mouthguard for cracks, tears, or signs of wear. If you notice any damage or if it no longer fits properly, schedule an appointment for a replacement.
- Avoid chewing: this can cause damage and reduce its effectiveness.
- Bring to appointments: bring your mouthguard to your regular dental appointments so your dentist can check its condition and fit.
- Replace as needed: mouthguards typically need replacement after a season or sooner if they show wear. Growth or orthodontic adjustments may also require a new fit.

## 4 Outcome and Follow‐Up

**FIGURE 11:**
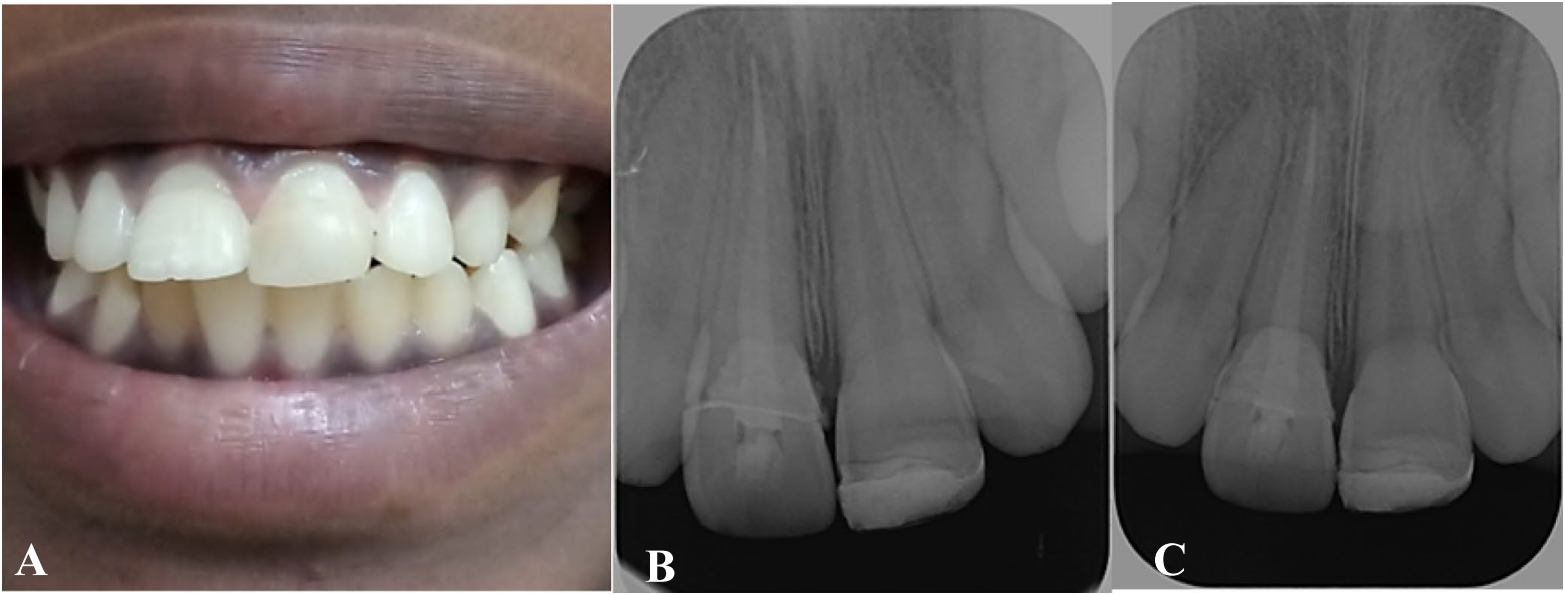
Post‐operative clinical photos and radiograph. (A) Frontal view after 12 months. (B) Periapical radiograph after 6 weeks. (C) Periapical radiograph after 12 months.

A follow‐up appointment was conducted after 2 weeks, followed by another after 6 weeks, at which point a periapical radiograph was obtained (Figure 11). Upon reassessment after 12 months, the examination revealed a palatal probing depth of 2 mm, absence of bleeding on probing, and normal tooth mobility. Additionally, the radiographic evaluation demonstrated stable reattachment of the fragments and maintained periodontal health (Figure 11).

Tooth fragment reattachment utilizing a fiber‐reinforced post in conjunction with the original tooth fragment represents a straightforward and effective modality for the treatment of fractured anterior teeth. This technique not only enhances aesthetic outcomes but also yields superior functional results.

## 5 Discussion

Traumatic injuries to anterior teeth are prevalent, impacting approximately 20%–48% of children under 18 years of age, with boys being nearly twice as likely to sustain these injuries compared to girls. This disparity can be attributed to boys' engagement in high‐risk activities, such as cycling and contact sports, whereas girls generally participate in less hazardous undertakings [9]. Recognizing these patterns allows dental professionals to implement targeted preventive measures and to devise effective management strategies [10, 11, 12].

Research indicates that leisure activities serve as a primary context for dental trauma, with nearly half of the injuries among children aged 7–18 occurring during these activities [13]. In younger children, falls are the leading cause of trauma, accounting for 89% of cases, while violence emerges as a significant contributor among older children, responsible for 42.5% of injuries within the 9–12 age group [11, 14]. Compromised aesthetics, rather than pain or functional impairments, remain the predominant reason for dental consultations following trauma, representing 48% of cases [14].

Crown fractures constitute the most common traumatic injuries to permanent teeth, comprising 26%–76% of cases, while crown‐root fractures are comparatively rare, accounting for 0.3%–5% [10, 15]. Uncomplicated fractures, which involve only enamel and dentin, are reported in 49% of cases, whereas complicated fractures, which necessitate pulpal treatment, occur in 8.5%–34.5% of instances [16, 17, 18]. Treatment modalities for such injuries include pulp therapy, restorative procedures, and various surgical interventions [10, 14].

### 5.1 Treatment Modalities for Crown‐Root Fractures

A range of techniques is available for managing crown‐root fractures, encompassing conservative approaches such as composite restorations and more invasive procedures like surgical extrusion or tooth extraction [19]. With advancements in dentin bonding agents and adhesive materials, fragment reattachment has emerged as a preferred method, providing strength comparable to that of intact teeth [20].

The success of fragment reattachment is contingent upon numerous factors, including the location of the fracture, the duration of time since the trauma occurred, pulpal involvement, and the type of materials and posts employed [21]. Advanced techniques, such as the use of fiber‐reinforced resin posts, enhance the retention and aesthetic outcomes of reattached fragments [22, 23, 24]. These posts possess a modulus of elasticity akin to that of dentin, facilitating improved stress distribution and reducing the likelihood of future fractures [25, 26, 27, 28].

### 5.2 Advantages of Fragment Reattachment

Fragment reattachment preserves natural tooth structure while restoring the original anatomical shape, color, and surface morphology. This minimally invasive and cost‐effective technique is also straightforward and time‐efficient, making it an ideal choice for managing dental trauma [7, 25, 26]. Additionally, fiber‐reinforced posts enhance the fracture resistance of restorations and ensure stability by allowing the post and tooth to function cohesively [28]. The use of resin cements further improves retention and minimizes microleakage, contributing to durable outcomes [29, 30, 31].

### 5.3 Challenges and Considerations

Clinical success necessitates a careful assessment of occlusal conditions, as unfavorable factors such as deep bite or bruxism may compromise the reattachment. Vertical root fractures, which can be caused by trauma, occlusal imbalances, or excessive masticatory forces, are contraindications for this treatment. Nevertheless, in favorable cases, reattachment has demonstrated excellent aesthetic and functional results, as confirmed in follow‐up evaluations [32].

Mouthguards are effective devices that decrease the likelihood of injuries to orofacial tissues and minimize the extent of damage [33]. The most frequently utilized materials for custom mouthguards include ethylene vinyl acetate (EVA), soft acrylic resin, polyvinyl chloride, polyvinyl acetate‐polyethylene, and elastomers [34, 35, 36, 37]. In the current study, the mouthguard was made from a thermoplastic EVA polymer. This material is recognized for its outstanding impact resistance, shock absorption, energy distribution, and ease of processing [38, 39].

### 5.4 Postoperative Care and Follow‐Up

Effective postoperative care is crucial for optimal healing and long‐term success. Patients should be advised on dietary modifications, pain management, and the necessity of regular follow‐ups, including radiographic evaluations to detect potential complications [4]. Long‐term follow‐up is essential to assess the durability and success of reattached teeth.

## Author Contributions

**Mohammed H. AbdElaziz:** conceptualization, methodology, supervision, writing – original draft. **Meshal Alharbi:** methodology, supervision, writing – original draft. **Maher O. Shahada:** investigation, writing – original draft. **Roqia Abdoh:** supervision, writing – original draft. **Radhwan Saleh Algabri:** writing – original draft, writing – review and editing. **Ahmed Yaseen Alqutaibi:** writing – original draft, writing – review and editing.

## Ethics Statement

The authors carefully examined all ethical concerns prior to publishing this case and its related images, ensuring that written informed consent from the parents was obtained.

## Consent

Written informed consent was obtained from the patient's father for publication of this case report. A copy of the consent form is available for the editor's review.

## Conflicts of Interest

The authors declare no conflicts of interest.

## Data Availability

The data and materials used in the current study are available from the corresponding author upon reasonable request.
